# Downregulation of the S1P Transporter Spinster Homology Protein 2 (Spns2) Exerts an Anti-Fibrotic and Anti-Inflammatory Effect in Human Renal Proximal Tubular Epithelial Cells

**DOI:** 10.3390/ijms19051498

**Published:** 2018-05-17

**Authors:** Olivier Blanchard, Bisera Stepanovska, Manuel Starck, Martin Erhardt, Isolde Römer, Dagmar Meyer zu Heringdorf, Josef Pfeilschifter, Uwe Zangemeister-Wittke, Andrea Huwiler

**Affiliations:** 1 Institute of Pharmacology, University of Bern, Inselspital, INO-F, CH-3010 Bern, Switzerland; olivier.blanchard@pki.unibe.ch (O.B.); bisera.stepanovska@pki.unibe.ch (B.S.); manuel.starck@pki.unibe.ch (M.S.); martin.erhardt@pki.unibe.ch (M.E.); uwe.zangemeister@pki.unibe.ch (U.Z.-W.); 2 Institute of General Pharmacology and Toxicology, University Hospital Frankfurt am Main, Goethe-University, Theodor-Stern Kai 7, D-60590 Frankfurt am Main, Germany; I.Roemer@em.uni-frankfurt.de (I.R.); heringdorf@med.uni-frankfurt.de (D.M.z.H.); Pfeilschifter@em.uni-frankfurt.de (J.P.)

**Keywords:** human renal proximal tubular epithelial cells, fibrosis, inflammation, sphingosine 1-phosphate, sphingosine kinase 1, spinster homology protein 2 (Spns2), CTGF, aquaporin 1

## Abstract

Sphingosine kinase (SK) catalyses the formation of sphingosine 1-phosphate (S1P), which acts as a key regulator of inflammatory and fibrotic reactions, mainly via S1P receptor activation. Here, we show that in the human renal proximal tubular epithelial cell line HK2, the profibrotic mediator transforming growth factor β (TGFβ) induces SK-1 mRNA and protein expression, and in parallel, it also upregulates the expression of the fibrotic markers connective tissue growth factor (CTGF) and fibronectin. Stable downregulation of SK-1 by RNAi resulted in the increased expression of CTGF, suggesting a suppressive effect of SK-1-derived intracellular S1P in the fibrotic process, which is lost when SK-1 is downregulated. In a further approach, the S1P transporter Spns2, which is known to export S1P and thereby reduces intracellular S1P levels, was stably downregulated in HK2 cells by RNAi. This treatment decreased TGFβ-induced CTGF and fibronectin expression, and it abolished the strong induction of the monocyte chemotactic protein 1 (MCP-1) by the pro-inflammatory cytokines tumor necrosis factor (TNF)α and interleukin (IL)-1β. Moreover, it enhanced the expression of aquaporin 1, which is an important water channel that is expressed in the proximal tubules, and reverted aquaporin 1 downregulation induced by IL-1β/TNFα. On the other hand, overexpression of a Spns2-GFP construct increased S1P secretion and it resulted in enhanced TGFβ-induced CTGF expression. In summary, our data demonstrate that in human renal proximal tubular epithelial cells, SK-1 downregulation accelerates an inflammatory and fibrotic reaction, whereas Spns2 downregulation has an opposite effect. We conclude that Spns2 represents a promising new target for the treatment of tubulointerstitial inflammation and fibrosis.

## 1. Introduction

Sphingosine 1-phosphate (S1P) is a central sphingolipid molecule that regulates a variety of important cellular responses, including proliferation and migration, and it also modulates inflammatory and fibrotic responses depending on its site of action [1,2,3]. Most of its reported actions are mediated by five identified high-affinity S1P receptor subtypes, which are denoted as S1P_1−5_ [4]. The receptor subtypes differentially couple to multiple G proteins, and thereby activate a complex network of signal transduction events and cellular outcomes. Alternatively, S1P can act as an intracellular second messenger and activate intracellular targets, which, however, are still poorly defined and controversial. Among these, the tumor necrosis factor (TNF) receptor-associated factor 2 (TRAF2) was shown to directly bind S1P, which activates its ubiquitin E3 ligase activity and leads to nuclear factor κB (NFκB) activation [5]. In addition, direct binding of S1P to nuclear histone deacetylase (HDAC)1 and HDAC2 [6], mitochondrial prohibitin-2 [7], and cytosolic ceramide synthase-2 [8] was reported.

S1P is generated by sphingosine kinases (SK), of which two subtypes SK-1 and SK-2 and several splice variants exist [9]. Both enzymes are ubiquitously expressed, but they differ slightly in their biochemical and enzymatic properties, and most importantly in subcellular localizations [9]. However, the physiological or pathophysiological function of these two enzymes, whether differential or similar, is currently not fully understood. Whereas, many reports have shown a role for SK-1 in the cell proliferation and migration of multiple cell types, the role of SK-2 is still controversial, ranging from pro-apoptotic [10], anti-proliferative [11,12], to even pro-proliferative [13] and pro-migratory [14].

As S1P is generated intracellularly (iS1P), it must be exported out of the cell to act as a receptor ligand. It has become clear that the transporter spinster homology protein 2 (Spns2) is mainly responsible for the secretion of S1P from many cell types [15]. Spns2 belongs to the major facilitator superfamily (MFS) of atypical solute carriers (SLC), and its gene deficiency in mice leads to a 40% reduction of plasma S1P levels [16] due to the lack of S1P release from vascular endothelial cells. Yet, in erythrocytes and platelets, Spns2 knockout had no effect on S1P secretion suggesting that other carriers exist in these cells, most notably the ABCA7 and ABCC1 transporters [17]. Spns2 deficient mice develop lymphopenia due to the reduced egress of mature T cells from the thymus to the blood, and further develop a profound hearing loss due to a defect in endocochlear potential and progressive degeneration of sensory hair cells in the organ of Corti [18]. The pathophysiological relevance of Spns2-mediated S1P secretion was recently reported by Donoviel et al. [19] who used Spns2-deficient mice in various disease models including airway inflammation and hypersensitivity, dextran sulfate sodium-induced colitis, collagen-induced arthritis, and experimental autoimmune encephalomyelitis, and described a strong reduction of disease parameters. Obviously, retaining S1P inside the cells has a protective effect in all of these inflammation associated models. So far, not much is known about the regulation of Spns2 expression or posttranslational modifications. Fu and colleagues recently reported that in human lung microvascular endothelial cells, hepatocyte growth factor can stimulate PI3K/Akt-dependent phosphorylation of Spns2 at a serine residue and trigger redistribution from a diffuse cytosolic location to the cell periphery, where it co-localizes with actin in lamellipodia [20].

Renal proximal tubular epithelial cells play an important role in tubulointerstitial inflammation and fibrosis. Proteinuria and protein overload to the tubular system, which develops in several forms of chronic kidney diseases, is considered to be a key factor that triggers the activation of proximal tubular epithelial cells to produce and/or secrete into the interstitium various inflammatory mediators, including interleukin (IL)-6, IL-8, monocyte chemoattractant peptide 1 (MCP-1/CCL2), RANTES/CCL5, and the intercellular adhesion molecule 1, but also fibrotic molecules such as connective tissue growth factor (CTGF) and fibronectin [21]. Many studies have shown that under cell culture conditions, proximal tubular epithelial cells are activated by the pro-fibrotic mediator transforming growth factor (TGF)β to undergo epithelial-mesenchymal transition (EMT) which is demonstrated by reduced expression of epithelial markers such as cytokeratin and E-cadherin, and increased the expression of mesenchymal markers, such as α-smooth muscle actin and vimentin. This suggests that tubular epithelial cells may also transform in vivo to myofibroblasts during fibrosis [22]. However, evidence for EMT in vivo in tubulointerstitial fibrosis is still controversial, and convincing evidence is still missing [23]. Proximal tubular epithelial cells also express a multitude of transporters that are important for the physiological function of the renal tubular system, i.e., reabsorption of solutes, peptides, and ions. In addition, various aquaporin (AQP) water channels are expressed along the tubular system and mediate water reuptake [24]. Under pathophysiological conditions, such as acute kidney injury, sepsis, and chronic inflammatory kidney diseases, the expression of AQPs is often altered, leading to either polyuria or to increased water reabsorption and edema formation. Notably, AQP-1, -2 and -3 were reported to be downregulated in experimental models of acute renal injury, and treatment strategies that reduced acute renal injury and polyuria also normalized AQPs expression [25]. However, the detailed mechanisms that especially regulate AQP1 are still unclear.

In this study, we investigated the effect of SK-1 knockdown (kd), which reduces iS1P, and of Spns2kd, which increases iS1P, on the fibrotic and the inflammatory reaction in the human renal proximal tubular epithelial cell line HK2. We show that, in HK2 cells, the profibrotic cytokine TGFβ upregulates CTGF and fibronectin expression, and in parallel also SK-1 expression. Upon SK-1 knockdown, CTGF was further increased, thus supporting our previous hypothesis based on data with other renal cell types that iS1P has a suppressive effect on fibrotic processes [1]. Spns2 knockdown was anti-fibrotic and anti-inflammatory, as it decreased TGFβ-stimulated expression of CTGF and fibronectin as well as expression of the pro-inflammatory chemokine MCP-1. These data underline the promise of Spns2 as an attractive pharmacological target to prevent secretion of iS1P and thereby dampen inflammatory and fibrotic responses, such as those seen in tubulointerstitial nephritis.

## 2. Results

**Figure 1:**
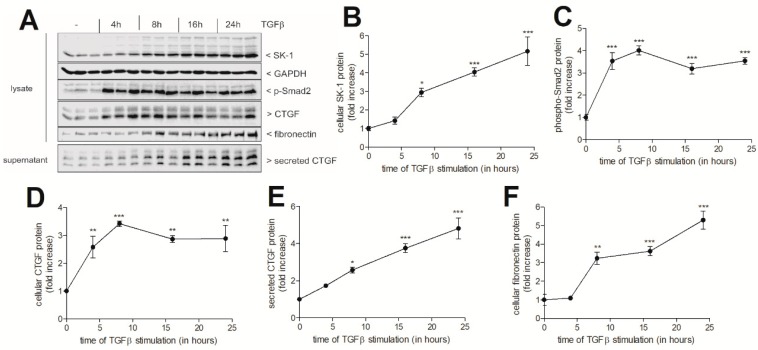
Effect of transforming growth factor β (TGFβ_2_) on SK-1, phospho-Smad2, connective tissue growth factor (CTGF), and fibronectin expression in HK2 cells. (**A**) HK2 cells were taken unstimulated (−) or stimulated with 5 ng/mL of TGFβ_2_ for the indicated time periods up to 24 h. Thereafter, cell lysates were prepared and equal amounts of proteins were separated by sodium dodecyl sulfate-polyacrylamide gel electrophoresis (SDS-PAGE), transferred to a nitrocellulose membrane and subjected to Western blotting using antibodies against SK-1 (1:2000 dilution), glyceraldehyde 3-phosphate dehydrogenase (GAPDH) (1:2000), phospho-Smad2 (1:1000), CTGF (1:500), and fibronectin (1:500). To detect secreted CTGF, equal volumes of supernatants of stimulated cells were taken for protein precipitation by trichloroacetic acid (TCA) and were then analysed the same way as described for lysates using the CTGF antibody. Band intensities were evaluated by a LICOR^R^ imaging system. Data in (**B**–**F**) show the evaluated respective bands. Results are expressed as fold increase and are means ± S.D. (*n* = 3); * *p* < 0.05, ** *p* < 0.01, *** *p* < 0.001 considered statistically significant when compared to the respective control values.

**Figure 2:**
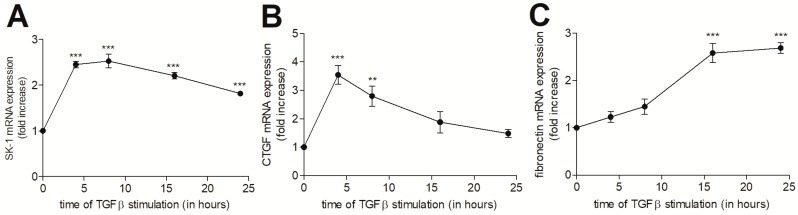
Time-dependent effect of TGFβ_2_ on the mRNA expression of SK-1, CTGF, and fibronectin in HK2 cells. Quiescent HK2 cells were stimulated with 5 ng/mL of TGFβ_2_ for the indicated time periods. Thereafter, RNA was extracted and taken for qPCR analysis using primers for SK-1 (**A**), CTGF (**B**), and fibronectin (**C**). Results are expressed as fold increase compared to the untreated control and are means ± S.D. (*n* = 3), ** *p* < 0.01, *** *p* < 0.001 considered to be statistically significant when compared to the respective control values.

To investigate the contribution of cellular S1P to tubulointerstitial inflammation and fibrosis, we used the human renal proximal tubular epithelial cell line HK2, which represents an important cell type that is involved in tubulointerstitial nephritis that are characterized by both an inflammatory and a fibrotic phase. In a first step, we treated HK2 cells with the pro-fibrotic mediator TGFβ and the pro-inflammatory cytokines TNFα and IL-1β to see whether the SK-1 protein expression is affected under fibrotic or inflammatory conditions. As seen in Figure 1A,B, TGFβ stimulated a time-dependent upregulation of SK-1 protein expression with a maximal effect after 24 h of treatment. Since TGFβ is well known to stimulate the Smad signalling cascade, which mediates gene transcription of various pro-fibrotic factors including CTGF and fibronectin, we analysed first whether the Smad signalling cascade is activated by TGFβ in HK2 cells by measuring Smad2 phosphorylation. Phospho-Smad2 was strongly increased at 4 h, and it remained high over 24 h (Figure 1A,C). In addition, CTGF protein was also upregulated by TGFβ in a time-dependent manner (Figure 1A,D,E). A similar upregulation was also detected for fibronectin (Figure 1A,F). The increased protein expressions of SK-1, CTGF, and fibronectin were preceded by increased mRNA expressions of SK-1 (Figure 2A), CTGF (Figure 2B), and fibronectin (Figure 2C).

**Figure 3:**
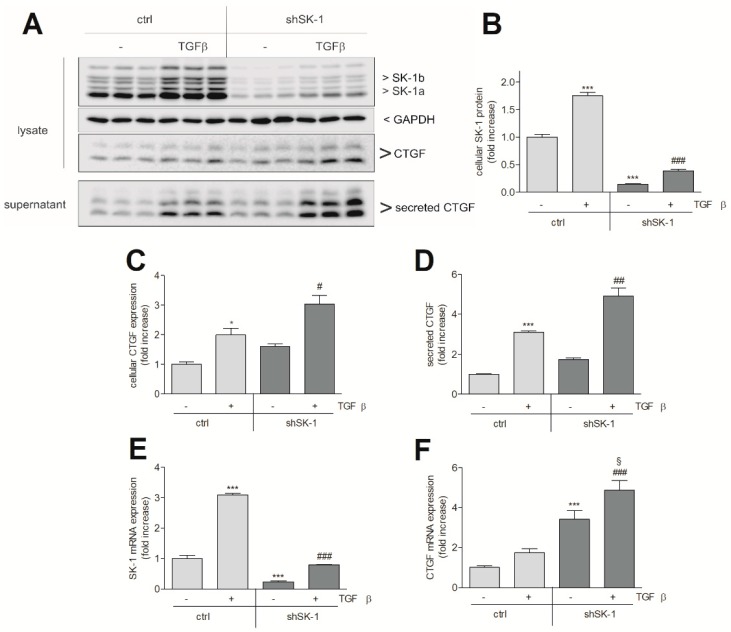
Effect of SK-1 knockdown on TGFβ-stimulated CTGF expression in HK2 cells. (**A**–**D**): HK2 cells stably transduced with either a lentiviral control vector (ctrl) or a SK-1 shRNA construct (shSK-1) were stimulated for 24 h with either vehicle (−) or 5 ng/mL of TGFβ_2_. Thereafter, cell lysates were taken for protein extraction and equal amounts of proteins were separated by SDS-PAGE, transferred to a nitrocellulose membrane, and then subjected to Western blotting using antibodies against SK-1, recognizing both splice variants SK-1a and -1b, CTGF, and GAPDH. To detect secreted CTGF, supernatants of stimulated cells were taken for protein precipitation by TCA and then analysed the same way, as described for lysates using the CTGF antibody. Band intensities were evaluated using a LICOR^R^ imaging system. (**E**,**F**): cells were stimulated for 4 h with either vehicle (−) or 5 ng/mL TGFβ_2_. Thereafter, RNA was extracted and taken for qPCR analysis using primers for SK-1 (**E**) and CTGF (**F**). Results are expressed as fold increase when compared to the vehicle-treated controls and are means ± S.D. (*n* = 3); * *p* < 0.05, *** *p* < 0.001 considered to be statistically significant when compared to the vehicle-treated control values; ^#^ *p* < 0.05, ^##^ *p* < 0.01, ^###^ *p <* 0.001 as compared to the TGFβ-treated control values; ^§^ *p* < 0.05 as compared to the vehicle-treated shSK-1 values.

**Figure 4:**
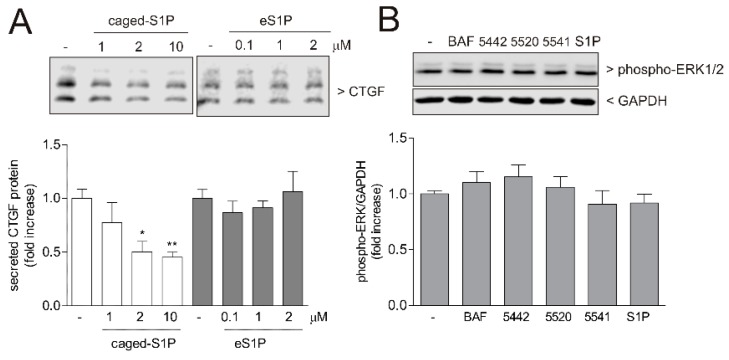
Effect of caged-S1P and extracellular S1P (eS1P) on CTGF expression and extracellular signal-regulated kinase (ERK) phosphorylation in HK2 cells. (**A**) Left panel: HK2 cells were treated with either vehicle (−), 1, 2 or 10 μM of caged S1P for 20 min for cellular uptake. Then cells were washed with PBS and illuminated for 30 s with light of 366 nm wavelength and further incubated for 24 h in DMEM. Right panel: Cells were stimulated for 24 h with 0.1, 1, or 2 μM S1P. (**B**) Cells were stimulated for 10 min with vehicle (−) or the different S1PR agonists: S1P_1 + 5_ agonist BAF312 (BAF, 1 μM), S1P_1_ agonist CYM5442 (5442, 1 μM), S1P_2_ agonist CYM5520 (5520, 10 μM), S1P_3_ agonist CYM5541 (5541, 2 μM), and S1P (2 μM). Thereafter, cell lysates or supernatants were taken for SDS-PAGE, transferred to a nitrocellulose membrane, and then subjected to Western blotting using antibodies against CTGF (**A**), phospho-ERK1/2 (**B**), and GAPDH (**B**). Bands were evaluated and results are depicted as fold increase and are means ± S.D. (*n* = 3); * *p* < 0.05, ** *p* < 0.01 considered to be statistically significant when compared to untreated controls.

As we had previously shown that in human glomerular podocytes, TGFβ-induced SK-1 acts as a brake in CTGF expression [26], we further studied here whether the same regulation occurs in HK2 cells. For this, a stable SK-1kd HK2 cell line was generated by the lentiviral transduction method. This cell line showed a 75–80% reduction of SK-1 protein (Figure 3A,B) and mRNA expression (Figure 3E). SK-1kd HK2 cells showed an increased basal CTGF expression and secretion (Figure 3C,D), which, upon TGFβ stimulation, was even further increased when compared to TGFβ-stimulated control HK2 cells. A similar effect was also seen for CTGF mRNA expression (Figure 3F). To test whether intracellular S1P indeed suppresses CTGF production, we used a caged-S1P, which does not bind S1P receptors, but is taken up by cells and upon illumination cleaves off the protective group generating active intracellular S1P [27,28]. When HK2 cells were treated with this caged-S1P, CTGF expression was concentration-dependently reduced (Figure 4A, left panel). As HK2 cells express several of the S1P receptors in the order S1P_1_ > S1P_5_ > S1P_2_ >> S1P_3_ (no S1P_4_), as measured by qPCR analysis, we also tested whether extracellular S1P has an effect on CTGF expression. However, S1P, used up to 2 μM, did not affect CTGF expression in these cells (Figure 4A, right panel), which contrasts the finding in other cell types, such as renal mesangial cells [29] and podocytes [26], which responded to extracellular S1P (eS1P) with increased CTGF production. Notably, eS1P and a series of more selective S1P receptor agonists, including BAF312 (S1P_1 + 5_), CYM5442 (S1P_1_), CYM5520 (S1P_2_), and CYM5541 (S1P_3_), were all unable to activate the extracellular signal-regulated kinase (ERK) signalling cascade (Figure 4B). These data suggest that, although various S1P receptors are expressed on the mRNA level, their functionality in HK2 cells is still unclear.

**Figure 5:**
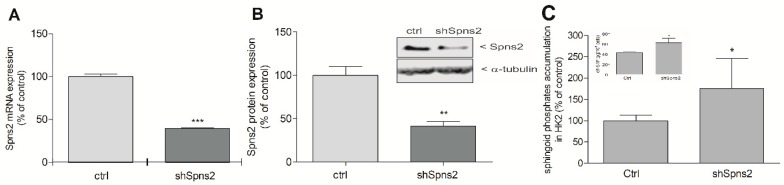
Characterization of Spns2 knockdown HK2 cells. HK2 cells stably transduced with either a lentiviral control construct (ctrl) or a Spns2 shRNA construct (shSpns2) were taken for RNA and protein extraction and analyzed by quantitative PCR and Western blotting for Spns2 mRNA (**A**) and protein expression (**B**). Cell monolayers of either control cells (Ctrl) or Spns2 knockdown cells (shSpns2) were lysed in PBS/high BSA and were taken for stimulation of S1P_3_-CHO cells (10 min) as described in the Methods Section, and ERK phosphorylation was determined by Western blotting (**C**). In parallel, cell monolayers were taken for lipid extraction and mass spectrometric quantification of sphingolipids. dhS1P (in pg/10^6^ cells) was the only sphingoid phosphate increased in shSpns2 (**C**, inset). Band intensities were evaluated using a LICOR^R^ imaging system. Results are expressed as % of control transduced cells and are means ± S.D. (*n* = 4–5 (**A**,**B**), *n* = 8 (**C**)); * *p* < 0.05, ** *p* < 0.01, *** *p* < 0.001 considered to be statistically significant when compared to the control vector samples.

**Figure 6:**
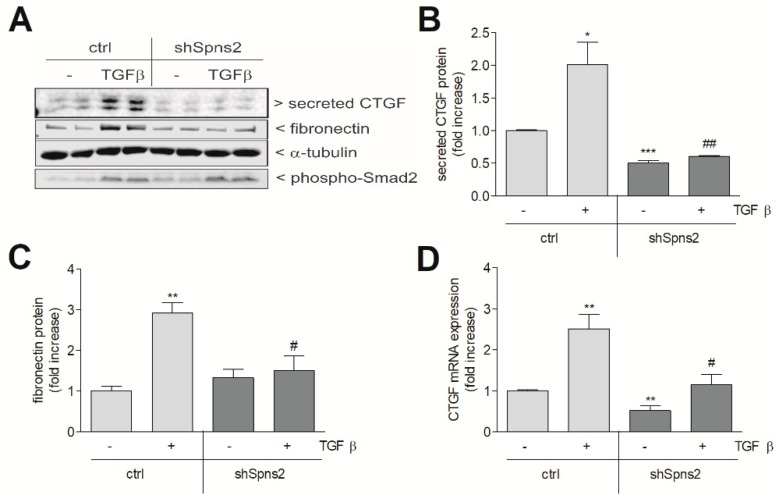
Effect of Spns2 knockdown on TGFβ-induced CTGF and fibronectin expression and Smad2 phosphorylation in HK2 cells. Control transduced cells (ctrl) and Spns2 knockdown cells (shSpns2) were stimulated for 20 h with either vehicle (−) or 5 ng/mL of TGFβ_2_. Thereafter, cell monolayers and supernatants were taken for protein extraction or precipitation, proteins were separated by SDS-PAGE, transferred to a nitrocellulose membrane, and then subjected to Western blotting using antibodies against CTGF (**A**), fibronectin (**A**), phospho-Smad2 (**A**), or α-tubulin (**A**). Band intensities were evaluated using a LICOR^R^ imaging system. Data in (**B**,**C**) show the evaluated respective bands from the Western blots, are expressed as fold increase, and are means ± S.D. (*n* = 3). (**D**) Cells were stimulated for 20 h with either vehicle (−) or TGFβ_2_ and RNA was extracted and taken for qPCR analysis using primers for CTGF. Results in D are expressed as fold increase and are means ± S.D. (*n* = 3); * *p* < 0.05, ** *p* < 0.01, *** *p* < 0.001 considered to be statistically significant when compared to the vehicle-treated control cells; ^#^ *p* < 0.05, ^##^ *p <* 0.01 as compared to the TGFβ-treated ctrl cells.

As our data suggest that iS1P, which derives from SK-1, has a suppressive effect on CTGF expression, we tested this possibility using an alternative strategy, i.e., by increasing iS1P by downregulating the S1P transporter Spns2. Spns2 is expressed in HK2 cells and lentiviral transduction of an Spns2 directed shRNA construct resulted in a stable Spns2kd HK2 cell line, which showed an approx. 60% reduction of Spns2 mRNA (Figure 5A) and protein (Figure 5B) when compared to control vector transduced cells. To determine the accumulation of S1P upon Spns2kd, an indirect reporter assay was used. In this assay, sonicated cell lysates were used to subsequently stimulate S1P_3_ receptor overexpressing CHO cells for 10 min and were analysed for ERK phosphorylation as an early read-out of receptor activation. As seen in Figure 5C, Spns2kd increased cellular sphingoid phosphates content as compared to control cells. Since the S1P_3_ receptor binds both S1P and dihydro-S1P, this assay cannot discriminate between the two species [30]. Therefore, the cellular levels of S1P and dihydro-S1P were quantitatively analysed by mass spectrometry. When compared to control cells, in Spns2kd cells only dihydro-S1P, which is generated directly by de-novo synthesis from dihydro-sphingosine upon SK action, but not S1P, was enhanced (Figure 5C inset). Notably, both of the species are transported by Spns2 [31] and show a similar S1P receptor activation profile [30]. Treatment of the Spns2kd cell line with TGFβ revealed a strongly reduced expression of CTGF protein (Figure 6A,B) and fibronectin protein (Figure 6A,C). Although the mRNA expression of CTGF was also reduced by Spns2kd (Figure 6D), phospho-Smad2 was not affected in TGFβ-stimulated Spns2kd cells as compared to TGFβ-stimulated control cells (Figure 6A, lowest panel). This suggests that the downregulation of CTGF gene transcription occurs independent of Smad activation.

**Figure 7:**
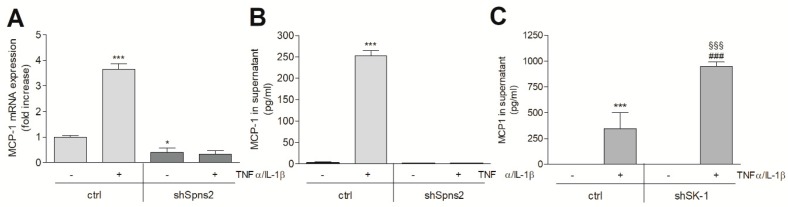
Effect of Spns2 knockdown and SK-1 knockdown on tumor necrosis factor (TNF)α/interleukin (IL)-1β-stimulated MCP-1/CCL2 mRNA and protein expression in HK2 cells. Control transduced cells (ctrl), Spns2 knockdown cells (shSpns2, **A**,**B**), or SK-1 knockdown cells (shSK-1, **C**) were stimulated for 20 h (**A**,**B**) or 24 h (**C**) with either vehicle or TNFα (1 nM) plus IL-1β (1 nM). Thereafter, either RNA was extracted and taken for qPCR analysis using primers of MCP-1 (**A**) or supernatants were collected and taken for a MCP-1 ELISA (**B**,**C**). Results in **A** are expressed as fold increase as compared to the vehicle-treated control cells and are means ± S.D. (*n* = 3). Results in (**B**,**C**) are expressed as pg/mL MCP-1 in the supernatant and are means ± S.D. (*n* = 3). * *p* < 0.05, *** *p* < 0.001 considered to be statistically significant when compared to the vehicle-treated control cells; ^###^ *p <* 0.001 as compared to the TNF/IL-1 stimulated ctrl cells; ^§§§^ *p* < 0.001 compared to the vehicle-treated shSK-1 cells.

Since HK2 cells are known to produce a variety of pro-inflammatory cytokines and chemokines under inflammatory conditions, we further investigated whether Spns2kd can affect this inflammatory response. MCP-1/CCL2 is well known to be strongly induced by proinflammatory stimuli [32,33], and it is considered to be a useful urinary biomarker for tubular inflammation and interstitial fibrosis [34]. In control HK2 cells, MCP-1 mRNA (Figure 7A) and protein expression (Figure 7B) were strongly induced by a combined TNFα plus IL-1β treatment, and this effect was abolished by Spns2kd (Figure 7A,B). In SK-1kd cells, the opposite effect was seen, i.e., MCP-1 protein showed enhanced expression when compared to the control cells (Figure 7C).

**Figure 8:**
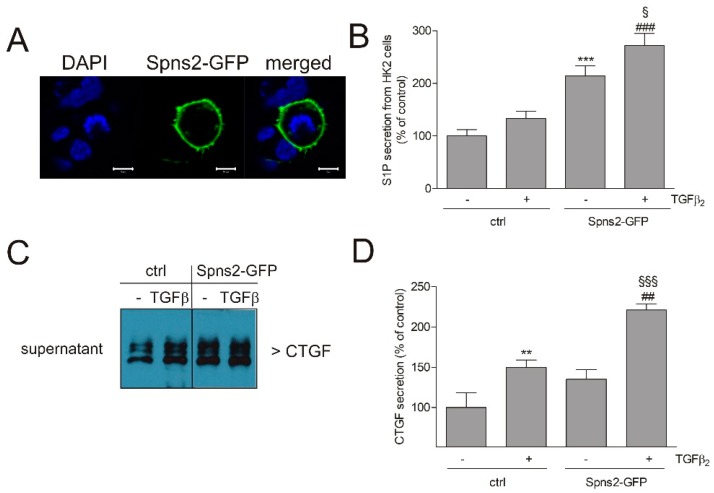
Effect of Spns2-GFP overexpression on localization, S1P release, and CTGF secretion in HK2 cells. Cells were transiently transfected with an empty GFP vector (Ctrl) or a Spns2-GFP vector. (**A**) Confocal picture 48 h post transfection, showing diamidine-phenylindole (DAPI) stained nuclei (blue), or Spns2-GFP (green), or a merged picture. Bars represent 10 μm. (**B**) Supernatants of ctrl cells or Spns2-GFP overexpressing cells, stimulated for 24 h with either vehicle (−) or TGFβ_2_ (5 ng/mL, +), were taken for a reporter assay to determine extracellular signal-regulated kinase (ERK) phosphorylation as a readout of S1P receptor activation, as described in the Methods Section. (**C**,**D**) Supernatants of vehicle (−) or TGFβ_2_ (+) stimulated cells were taken for protein precipitation, and proteins were separated by SDS-PAGE, transferred to a nitrocellulose membrane, and then subjected to Western blotting using antibodies against CTGF. Bands were densitometrically evaluated. Data are expressed as % of control and are means ± S.D. (*n* = 3–4); ** *p* < 0.01, *** *p* < 0.001 compared to the vehicle-treated ctrl cells; ^##^ *p* < 0.01, ^###^ *p* < 0.001 compared to the Spns2-GFP control; ^§^ *p* < 0.05, ^§§§^ *p* < 0.001 as compared to the TGFβ-stimulated control cells.

In a further approach, we overexpressed green fluorescence protein (GFP)-fused Spns2 in HK2 cells. Characterization of these transiently overexpressing cells by confocal microscopy revealed clear plasma membrane localization, as expected for the S1P-transporter (Figure 8A). Spns2 overexpressing cells also secreted more sphingoid phosphates, as determined by an indirect reporter assay (Figure 8B). Furthermore, basal and TGFβ-induced CTGF secretion was enhanced (Figure 8C,D). 

**Figure 9:**
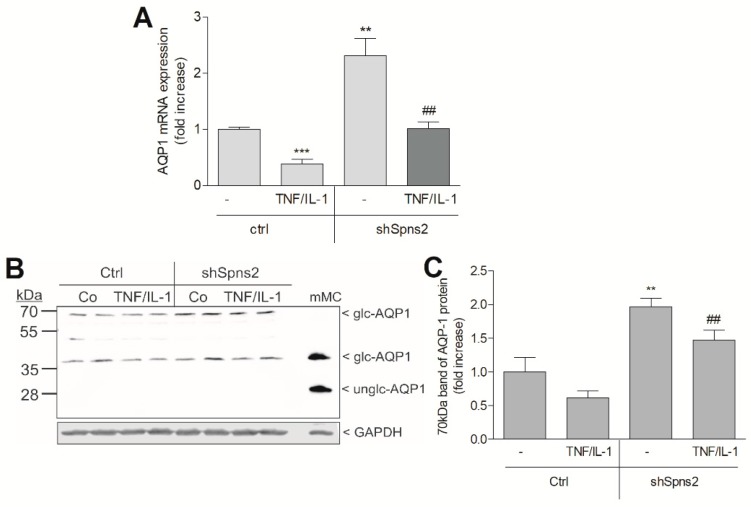
Effect of Spns2 knockdown on aquaporin 1 mRNA and protein expression in HK2 cells. Control (ctrl) or Spns2 knockdown cells (shSpns2) were stimulated for 20 h with vehicle (−) or TNFα (1 nM) plus IL-1β (1 nM). Thereafter, cells were taken for RNA extraction and subsequent qPCR analysis of aquaporin (AQP1) (**A**), or for protein extraction and Western blotting using antibodies against AQP-1 and GAPDH (**B**). The 70 kDa band of AQP1 was densitometrically evaluated (**C**). In B, mouse mesangial cells (mMC) were used as a standard for unglycosylated (28 kDa) and low glycosylated (38 kDa) AQP1. Results in A and C are expressed as fold increase as compared to controls and are means ± S.D. (*n* = 3–4); ** *p* < 0.01, *** *p* < 0.001 considered to be statistically significant when compared to the vehicle-treated ctrl cells, ^##^ *p* < 0.01 as compared to the TNFα/IL-1β-treated ctrl cells. Results in B show one representative blot with duplicate samples.

We next analyzed whether the main AQP expressed in the proximal tubular cells, i.e., AQP1, is affected by Spns2kd/S1P. As seen in Figure 9A, AQP1 mRNA expression in HK2 cells was downregulated by a combined TNFα plus IL-1β treatment. Spns2kd in HK2 cells resulted in increased basal AQP1 mRNA and the TNFα/IL-1β-mediated downregulation of AQP1 was normalized, suggesting, for the first time, a regulation of AQP1 by the S1P signalling system. A similar regulation was also seen on the protein level detected by Western blotting. AQP1 has been described as a 28 kDa protein that upon glycosylation appears in two higher molecular forms of 38–50 kDa and 55–70 kDa [35,36,37]. In HK2 cells, AQP1 ran as exclusively glycosylated forms of 38 kDa and 70 kDa with no detectable unglycosylated form (Figure 9B). Upon TNFα/IL-1β stimulation, the expression of the 38 kDa and 70 kDa AQP forms were partially downregulated. Notably, in Spns2kd cells, basal expression of the 70 kDa AQP1 was already enhanced and the downregulation by TNFα/IL-1β was less efficient (Figure 9B,C).

## 3. Discussion

In this study, we show for the first time that the S1P transporter Spns2 is expressed in renal proximal tubular epithelial cells where it regulates a fibrotic reaction (Figure 5A,B). Our data demonstrate that stable downregulation of Spns2 in HK2 cells protects these cells from TGFβ-induced CTGF and fibronectin expression (Figure 6), which are important pro-fibrotic mediators and fibrotic markers of tubulointerstitial fibrosis [38]. It suggests that iS1P, which is supposed to accumulate when Spns2 expression is reduced, can counter-regulate CTGF expression, a hypothesis that is further supported by our finding that caged-S1P, which upon illumination liberates active S1P in the intracellular space, reduces CTGF (Figure 4A). These data are in line with our previous study in glomerular podocytes, which were also protected by iS1P from CTGF expression [26]. However, in podocytes, we identified a profibrotic effect of eS1P, as it upregulated CTGF expression. This pro-fibrotic effect of eS1P could not be seen in tubular epithelial cells since eS1P could neither upregulate CTGF expression (Figure 4A) nor activate early ERK signalling (Figure 4B). We therefore hypothesize that S1P receptors, although expressed on the mRNA level, are not functional, maybe because they are stably desensitized or are not localized on the cell surface. Notably, proximal tubular epithelial cells are highly polarized cells and the expression pattern of receptor and transporters in vivo may be unique at either the apical or basolateral side. Furthermore, it is possible that secreted S1P acts in a paracrine manner on other cell types, such as fibroblasts/myofibroblasts in the interstitium, to promote the fibrotic events that are contributing to tubulointerstitial fibrosis.

Another factor that is strongly downregulated by Spns2kd in HK2 cells in our study is MCP-1/CCL2 (Figure 7). MCP-1/CCL2 is highly induced by inflammatory stimuli, like TNFα and IL-1β [39]. It is not only produced and secreted by monocytes and macrophages, but also by various renal cell types, such as glomerular mesangial cells, podocytes, and tubular epithelial cells [40,41,42]. Moreover, it is considered to be crucial for recruiting monocytes/macrophages to the kidney in many forms of inflammatory kidney diseases, thereby driving the progression of renal injury. Thus, not surprisingly, urinary MCP-1 has been suggested as a diagnostic marker for progressive renal injury in diabetic nephropathy [34,42], and pharmacological targeting of MCP-1 and its receptor CCR2 has been proposed as a novel approach to treat diabetic kidney disease [43,44]. Our data show that in SK-1kd cells, which have reduced iS1P levels, MCP-1 synthesis (Figure 7C), but also CTGF expression (Figure 3A) are enhanced, suggesting increased inflammation and fibrosis. These data are in agreement with previous reports on SK-1 knockout mice or mice that were treated with a selective SK-1 inhibitor, showing enhanced UUO-induced tubulointerstitial fibrosis [45] and streptozotocin-induced glomerulosclerosis by the loss of SK-1 activity [26].

In Spns2kd cells, MCP-1 induction is not only abolished on the protein level but also on the mRNA level, indicating that accumulating iS1P suppresses gene transcription of MCP-1. In this regard, the regulation of MCP-1 by S1P is still controversial. Chen et al. [46] showed that in human endothelial cells in culture, the downregulation of SK-1 using siRNA reduced TNFα-induced MCP-1 expression, suggesting a positive regulatory role of SK-1-derived S1P in MCP-1 expression. In agreement with these findings, a previous study on mouse peritoneal macrophages also showed that biglycan-triggered MCP-1/CCL2 production was strongly reduced in macrophages that were isolated from SK-1 knockout mice [47]. Similarly, in the lung cancer cell line A549, thrombin stimulated upregulation of SK-1 and MCP-1 expression, and downregulation of SK-1 using siRNA abolished thrombin-induced MCP-1 expression [48]. However, in a rat model of albumin overload-induced nephropathy, which typically showed increased MCP-1 production in the kidney, the treatment of rats with the functional S1P_1_ antagonist FTY720 decreased MCP-1 production and mitigated pathological features [49]. The mechanism of FTY720 mediated MCP-1 downregulation is still unclear since FTY720 can act in a dual manner, either as a short-term unselective S1P receptor agonist, or, when being applied in a sustained manner, as a selective functional antagonist of S1P_1_.

Gene transcription of MCP-1 is strongly driven by the transcription factors NFκB and Sp1, and the functional binding sites for these transcription factors have been identified in the promoter sequence of MCP-1 [50]. Whether iS1P affects MCP-1 transcription by interfering with one of these transcription factors remains to be determined. Especially, the role of S1P in inflammation is still a matter of debate. At least some studies have suggested a rather pro-inflammatory role of iS1P by activating NFκB, possibly through direct TRAF2 activation [5,47]. On the other hand, in other studies, macrophages that were isolated from myeloid SK-1/SK-2 double knockout mice showed no change of LPS-triggered NFκB activation or cytokine production, nor a change in proliferation or apoptosis, but instead showed increased autophagic markers [51]. In addition, Etemadi et al. [52] showed that neither TRAF2 nor SK-1 are required for TNFα-mediated canonical NFκB signalling in macrophages, whereas mouse embryonic fibroblasts, murine dermal fibroblasts, and keratinocytes required TRAF2, but not SK-1, for full strength TNF signalling and NFκB activation.

The role of Spns2 in inflammation is slowly beginning to emerge. Mainly the fact that blood S1P levels are reduced by 40% in Spns2-deficient mice [16] and that these mice are lymphopenic suggests that T lymphocyte-driven inflammatory and autoimmune diseases can in principle be reduced by Spns2 downregulation or inhibition. Indeed, it was recently shown that Spns2-deficient mice are protected in an airway inflammation and hypersensitivity model as well as in models of colitis, arthritis, and experimental autoimmune encephalomyelitis [19]. Interestingly, Sato et al. [53] recently reported that in patients with advanced liver fibrosis, mRNA expression of Spns2 was enhanced and correlated with serum alanine aminotransferase (ALT) levels. This upregulated Spns2 in liver fibrosis may result in enhanced S1P secretion and enhanced autocrine or paracrine action via S1P receptors to stimulate the fibrotic process.

S1P has been suggested to be critically involved in various models of acute tubular injury. In this regard, ischemia/reperfusion-induced liver injury in mice that leads not only to liver injury, but also to acute kidney injury, was accompanied by a reduction of plasma S1P and dihydro-S1P levels, and application of either S1P or dihydro-S1P acting through S1P_1_ was shown to reduce systemic inflammation and protect mice from liver and kidney injury [54,55]. Furthermore, in a mouse model of cisplatin-induced tubular injury, S1P and FTY720, via S1P_1_ activation, reduced acute kidney injury [56], which was suggested to be due to a stabilizing role of S1P_1_ in tubular mitochondrial function. Very recently, it was reported that SK-2 deficient mice develop less kidney fibrosis due to increased interferon (IFN)-γ production [57]. How exactly the loss of SK-2 leads to increased INFγ production was not further investigated. Similar data were also shown by Schwalm et al. [58] who demonstrated a protective effect of SK-2 deficiency in unilateral ureter-induced kidney fibrosis, which was mediated by the upregulation of the anti-fibrotic Smad7 in kidney tissue, but also seen in isolated SK-2-deficient renal fibroblasts and in SK-2 knockdown HK2 cells. Notably, SK-2-deficient mice are known to have increased S1P levels in the plasma [59] and in certain organs and cellular systems [60,61]. It is therefore tempting to speculate that the increased S1P, either intracellularly or extracellularly, somehow contributes to the protective effect. Furthermore, it was reported that nuclear S1P, as provided by SK-2, could inhibit HDAC activity [6], and HDAC inhibitors are known to reduce renal fibrosis [62]. Thus, it is possible that such a mechanism accounts for the protective effect seen in SK-2 deficient mice that are exposed to ureter ligation-induced interstitial fibrosis [57].

Our data also demonstrate for the first time that Spns2kd HK2 cells express higher levels of AQP1 mRNA and that AQP1 is decreased under inflammatory conditions, which is normalized by Spns2kd (Figure 9A). The proximal tubulus is highly equipped with multiple channels and transporters that are crucial for the physiological function of the renal tubulus, which includes the reabsorption of solutes, peptides, ions, and water. About 60% of the glomerular filtrate water reabsorption is handled by the proximal tubular system, and this is mainly driven by AQP1, which is abundantly expressed at this site. In many forms of renal disease, a reduced water reabsorption in the kidney is manifested, which correlates with reduced expression of various AQPs, including AQP1 [24,25], and AQP1-deficient mice typically show polyuria as they are unable to concentrate their primary urine [63]. Interestingly, these AQP1-deficient mice showed increased LPS-induced renal injury [64]. Additionally, it is worth noting that in LPS-induced sepsis in mice, plasma S1P levels are reduced [65,66]. These studies may indeed suggest an inverse correlation between the two effector molecules S1P and AQP1 and disease severity in systemic inflammation. So far, little is known about the transcriptional regulation of AQP1. It was previously shown to be transcriptionally upregulated by transferrin, which is a known proteinuric component perturbing tubular function [67], by d-glucose [68], hypertonic stress [69], and by PPARγ [68]. In addition, AQP1 transcription was shown to be regulated by the transcription factor MEF2c and Sp1 in endothelial cells, and to contribute to endothelial cell migration and tube formation [70]. Notably, S1P was also shown to increase the expression of MEF2c in cardiac progenitor cells [71], which makes it tempting to speculate that S1P regulates AQP1 via MEF2c as a general mechanism. How this is regulated in HK2 cells remains to be investigated.

Altogether, our data demonstrate that Spns2 and S1P have a crucial effect on proximal tubular epithelial cells by regulating an inflammatory process and a subsequent fibrotic reaction, and also by influencing other tubular transporters that are involved in the pathophysiology of inflammatory and fibrotic kidney diseases. We conclude that Spns2 represents a promising pharmacological target to treat these pathological conditions.

## 4. Materials and Methods

### 4.1. Chemicals

S1P and all C17-sphingolipid standards were from Avanti Polar Lipids Inc. (Alabaster, AL, USA); caged-S1P was from Enzo Life Sciences Inc. (Farmingdale, NY, USA); human TGF-β_2_, TNFα, and IL-1β were from Peprotech (London, UK); BAF312 was from Selleck Chemicals Inc. (Houston, TX, USA); CYM5442, CYM5520, CYM5541, puromycin, the lentiviral particles of human Spns2 and SK-1 shRNA constructs (MISSION^®^ shRNA), diamidine-phenylindole (DAPI), and the antibodies against α-tubulin and Spns2 (SAB2104271) were from Sigma Aldrich Chemikalien, (Buchs, Switzerland); antibodies against phospho-ERK1/2, phospho-Smad2, and glyceraldehyde 3-phosphate dehydrogenase (GAPDH) were from Cell Signaling Technology (Danvers, MA, USA); antibodies against CTGF (L-20) and fibronectin (EP5) were from Santa Cruz (Heidelberg, Germany); the AQP1 antibody (EPR11588(B)) was from Abcam plc (Cambridge, UK); the human SK-1 antibody was generated and characterized, as previously described [72,73]. The secondary anti-rabbit and anti-mouse horseradish peroxidase-coupled IgG antibodies and the enhanced chemiluminescence (ECL) reagents were from GE Health Care Systems GmbH (Freiburg, Germany). The human MCP-1 ELISA was from Boster Biological Technology (Pleasanton, CA, USA). All of the cell culture nutrients were from Life Technologies AG (Basel, Switzerland).

### 4.2. Cell Culture and Transfections

The human renal proximal tubular epithelial cell line HK2 (ATCC, American Type Culture Collection, Manassas, VA, USA) was cultured in DMEM/F-12 medium that was supplemented with 10% fetal bovine serum (FBS), 100 units/mL penicillin, and 100 μg/mL streptomycin at 37 °C in 5% CO_2_ atmosphere. Prior to stimulation, cells were serum starved by incubation for 16 h in Dulbecco’s modified Eagle medium (DMEM) including 0.1 mg/mL of fatty acid-free bovine serum albumin (BSA) and penicillin/streptomycin. For Spns2 knockdown experiments, 1% FBS was added to the starving medium. For stable gene silencing of SK-1 (SK-1kd) and Spns2 (Spns2kd), commercially available short hairpin RNA (shRNA) lentiviral transduction particles (MISSION^R^) were used. Transduction of HK2 cells was performed, according to the manufacturer’s instructions. SK-1kd HK2 cells were generated with lentiviral transduction particles, as previously described [74]. For Spns2kd HK2 cells, five different shRNA constructs were tested and the clone showing the strongest downregulation of Spns2 expression was used for further experiments. For stable selection, 1 μg/mL puromycin was included in the medium. For Spns2 overexpression, a GFP-fused cDNA of Spns2 (kindly provided by Dr. A. Kawahara, National Cardiovascular Center Research Institute, Osaka, Japan, [75]) was transiently transfected in HK2 wildtype cells using lipofectamin, as recommended by the manufacturer (Thermo Fisher Scientific Inc., Waltham, MA, USA) 48 h post transfection, cells were subcultured and taken for experiments or characterization of successful transfection by confocal microscopy. Chinese hamster ovary cells (CHO-S1P_3_) were kindly provided by Dr. D. Guerini (Novartis Pharma Inc., Basel, Switzerland) and cultivated in RPMI 1640 supplemented with 10% FBS, 50 μg/mL gentamycin and 0.5 mg/mL G418.

### 4.3. Western Blot Analysis

From stimulated cells the medium was collected for protein precipitation by adding trichloroacetic acid (TCA) up to a final concentration of 7% (*w*/*v*). Samples were incubated for 30 min at 4 °C and were centrifuged for 30 min at 13,000× *g*. Precipitated proteins were redissolved in SDS-Laemmli buffer and were taken for protein separation by sodium dodecyl sulfate-polyacrylamide gel electrophoresis (SDS-PAGE), protein transfer to nitrocellulose membrane, and Western blotting. Cell monolayers were washed once with ice-cold phosphate-buffered saline (PBS) solution. Thereafter, cells were scraped into ice-cold lysis buffer (50 mM Tris/HCl, pH 7.4, 150 mM NaCl, 10% glycerol, 1% Triton X-100, 2 mM EDTA, 2 mM EGTA, 40 mM β-glycerophosphate, 50 mM sodium fluoride, 10 mM sodium pyrophosphate, 2 mM dl-dithiothreitol, 200 μM sodium orthovanadate, 10 μg/mL leupeptin, 10 μg/mL pepstatin A, and 1 mM phenylmethyl sulphonyl fluoride) and were homogenized by sonication. The samples were then centrifuged for 10 min at 13,000× *g* and the supernatant was taken for protein determination. Cell lysates containing equal amounts of protein (35 μg) were separated by SDS-PAGE, transferred to nitrocellulose membrane for 1 h at 250 mA using a Transblot apparatus (Bio-Rad Laboratories GmbH, München, Germany). The blotting buffer used was 25 mM Tris, 190 mM glycine and 20% MeOH (*v*/*v*). After transfer, the nitrocellulose membranes were washed in distilled water and were blocked in blocking buffer containing 3% (*w*/*v*) low-fat milk powder in PBS for 1 h at 25 °C. Membranes were then incubated with the indicated antibodies overnight at 4 °C on a plate shaker. All of the antibodies were diluted in 50 mM Tris/HCl pH 7.4, 200 mM NaCl, 10% horse serum, 3% (*w*/*v*) BSA fraction V, 0.1% (*v*/*v*) Tween-20. Thereafter, membranes were washed 3 × 10 min in buffer containing 50 mM Tris/HCl pH 7.4, 200 mM NaCl, 0.2% (*v*/*v*) Triton X-100 and incubated with secondary antibodies for 1 h at 25 °C, washed 3 × 10 min, and then incubated with enhanced chemiluminescence (ECL) reagents, according to the manufacturers recommendations. For fibronectin transfer to nitrocellulose, 0.1% (*w*/*v*) SDS was added to the transfer buffer.

### 4.4. Quantitative Real-Time PCR Analysis (qPCR)

qPCR was performed using SYBRGreen^R^ and a Bio-Rad iQ iCycler Detection System. Primers were as follows: human SK-1: forward: 5′-GCT TCC TTG AAC CAT TAT G-3′; reverse: 5′-TCT CTA GGT CCA CAT CAG-3′; human CTGF: forward: 5′-TGC CTG CCA TTA CAA CTG TCC-3′, reverse: 5′-GCC ATG TCT CCG TAC ATC TTC C-3′; human fibronectin: forward: 5′-CGA AAT CAC AGC CAG TAG-3′; reverse: 5′-ATC ACA TCC ACA CGG TAG-3′; human Spns2: forward: 5′-ACT TTG GGG TCA AGG ACC GA-3′; reverse: 5′-AAT CAC CTT CCT GTT GAA GCG-3′; human MCP-1: forward: 5′-CCC CAG TCA CCT GCT GTT AT-3′; reverse: 5′-TGG AAT CCT GAA CCC ACT TC-3′; human aquaporin 1: forward: 5′-TGG ACA CCT CCT GGC TAT TG-3′; reverse: 5′-GGG CCA GGA TGA AGT CGT AG-3′; 18S RNA: forward: 5′-CGA TTC CGT GGG TGG TGG TG-3′; reverse: 5′-CAT GCC AGA GTC TCG TTC GTT ATC-3′. 1 μg of total RNA isolated with TRIzol^R^ (Thermo Fisher Scientific) reagent was used for reverse transcriptase cDNA synthesis (First Strand Synthesis Kit, Thermo Fisher Scientific); a random hexamer primer was used for amplification. Real-time fluorescence from SYBR^R^ Green (Sigma Aldrich) was measured by the Bio-Rad CFX Manager 3.1 System Software. The fold induction values were obtained, according to the ΔΔ*C*_t_ method, after normalization to the housekeeping gene 18S RNA. 

### 4.5. Determination of S1P Generation and Secretion by a Reporter Assay

HK2 cells were seeded onto 12-well plates and stimulated, as described in DMEM supplemented with 10 mg/mL of BSA. In parallel, the cells in additional wells were counted to determine cell number. After stimulation, HK2 cell monolayers were washed once with ice-cold PBS, scraped into PBS containing 10 mg/mL BSA and homogenized by sonication. Samples were centrifuged for 10 min at 13,000× *g* at 4 °C and the supernatant was stored at −80 °C for further CHO-S1P_3_ cell stimulations to determine iS1P concentrations. In addition, media from stimulated HK2 cells were collected, centrifuged for 5 min at 1500× *g* at 4 °C, and the supernatants were collected for further CHO-S1P_3_ stimulation using 10% (*v*/*v*) of the conditioned medium in fresh DMEM. Prior to stimulation, CHO-S1P_3_ cells were starved for 22 h in serum-free DMEM, including 0.1 mg/mL of fatty acid-free BSA and then stimulated for 10 min with either DMEM containing the HK2 lysate (5000 cell equivalents per mL), or with 10% (*v*/*v*) HK2 conditioned medium. Thereafter, CHO-S1P_3_ cells were washed with PBS and then harvested for Western blotting to determine ERK phosphorylation as a read-out for receptor activation.

### 4.6. Quantification of S1P by Mass Spectrometry

Cell monolayers in 35 mm-diameter dishes were trypsinized, pelleted and stored at −80 °C until further processing. Samples were resuspended in methanol containing internal C17-ceramide, C17-sphingosine, and C17-S1P standards, and were subjected to lipid extraction and LC/MS/MS analysis, as previously described [76].

### 4.7. Statistical Analysis

Statistical analysis was performed by one-way analysis of variance (ANOVA). For multiple comparisons with the same control group, the limit of significance was divided by the number of comparisons, according to Bonferroni.

## Abbreviations

AQP	aquaporin
BSA	bovine serum albumin
CHO cells	Chinese hamster ovary cells
CTGF	connective tissue growth factor
DMEM	Dulbecco’s modified Eagle medium
ECL	enhanced chemiluminescence
ERK	extracellular signal regulated kinase
eS1P	extracellular sphingosine 1-phosphate
FBS	fetal bovine serum
GAPDH	glyceraldehyde 3-phosphate dehydrogenase
GFP	green fluorescence protein
HDAC	histone deacetylase
HK2	human proximal tubular epithelial cells
IFN	interferon
IL	interleukin
iS1P	intracellular S1P
kd	knockdown
MCP-1/CCL2	monocyte chemoattractant peptide 1
MFS	major facilitator superfamily
NFκB	nuclear factor κB
SDS-PAGE	sodium dodecyl sulfate-polyacrylamide gel electrophoresis
shRNA	small hairpin RNA
SK	sphingosine kinase
Spns2	spinster homology protein 2
TGFβ	transforming growth factor β
TNFα	tumor necrosis factor α
TRAF2	TNF receptor-associated factor 2
UUO	unilateral ureter obstruction
